# Drug Adherence After Hospitalisation for Heart Failure: What Have We Learned from a French Survey?

**DOI:** 10.3390/jcm15093483

**Published:** 2026-05-02

**Authors:** Aurélie Lenglet, Emmanuelle Vermes, Maxime Doublet, Richard Isnard, François Roubille, Thibaud Damy, Christophe Tribouilloy, Damien Logeart

**Affiliations:** 1 Hospital Pharmacy, Jules Verne University of Picardie, 80000 Amiens, France; terrier-lenglet.aurelie@chu-amiens.fr (A.L.); tribouilloy.christophe@chu-amiens.fr (C.T.); 2 Cardiology Department, University Hospital Amiens-Picardie, 80054 Amiens, France; vermes.emmanuelle@chu-amiens.fr; 3 Clinityx, 75008 Boulogne-Billancourt, France; 4 Pitié–Salpêtrière Hospital, Assistance Publique-Hôpitaux de Paris, 75013 Paris, France; richard.isnard@aphp.fr; 5 PhyMedExp, Cardiology Department, University of Montpellier, 34295 Montpellier, France; 6 Henri Mondor Hospital, Assistance Publique-Hôpitaux de Paris, 94000 Créteil, France; 7 Lariboisière Hospital, Assistance Publique-Hôpitaux de Paris, 75010 Paris, France; 8 Department of Cardiology, Paris Cité University, 75010 Paris, France

**Keywords:** heart failure, drug adherence

## Abstract

**Background:** Despite significant advances in heart failure (HF) management, mortality and readmission rates remain persistently high. Low adherence has been recognised as a contributing factor, although supporting data remain limited. Objective: This study aimed to evaluate the impact of medication adherence on outcome following a HF hospitalisation. **Methods:** Patients who were discharged after HF hospitalisation were included in the study from a national multicentre HF cohort, and their records were matched with the National Healthcare System database, which includes all health-related claims and clinical events. Adherence to beta blockers, renin-angiotensin system inhibitors, and mineralocorticoid receptor antagonists were measured using the proportion of days covered (PDC). Low adherence was defined by PDC < 80% for at least one of the three HF drug classes. We then analysed the relationship between the PDC and outcome during a two-year follow-up period. **Results:** A total of 448 patients (median age: 73 years; 67% male; mean ejection fraction: 40%) were included. Of these patients, 152 (34%) were classified as having low adherence. The two-year mortality rate was comparable between the two groups (16.9% vs. 19.1% in adherent and low-adherent groups, respectively, *p* = 0.6). However, the rates of all-cause and HF rehospitalisations at two years were lower in the adherent group than in the group with low adherence (85.9% vs. 92.8%, *p* ≤ 0.01; 48.5% vs. 58.2%, *p* = 0.04, respectively). **Conclusions**: In patients discharged after acute HF, low adherence to HF drugs is frequent and worsens outcome, particularly the risk of rehospitalisation.

## 1. Introduction

Heart failure (HF) is a prevalent and life-threatening condition affecting more than 64 million people worldwide [1]. The global burden of HF is significant, with a prevalence of 1–3% in the adult population and short-term (30 days) and long term (5 years) mortality rates of 2–3% and 50–75% respectively. HF also incurs expensive annual healthcare costs [2].

Significant advancements in HF management have been achieved through the development of device therapies and medications that decrease this burden [3]. HF drugs and their combinations have been proven in randomised clinical trials, including highly selected patient populations, to reduce symptoms and to decrease the rate of hospitalisation and death [4,5,6,7,8,9,10]. However, in actual clinical practice, many patients do not receive optimal HF therapies [11,12,13]. In addition to medical inertia, non-adherence to HF medication significantly increase morbidity, mortality and healthcare costs [14,15]. The effectiveness of treatments depends on adherence to medical advice, which is defined as taking the recommended dosage, frequency and duration of drug regimens prescribed by healthcare providers. Adherence to medications is a process comprising initiation, implementation and discontinuation [16]. Non-adherence to medications can thus occur when treatment is not initiated, when the dosing regimen is not followed optimally, or when treatment is discontinued prematurely. One common method of measuring medication adherence is through pharmacy refill data which provides objective evidence based on the timing of prescription refills. Various measures have been developed to calculate refill adherence from administrative data such as pharmacy claims databases [17]. One of the most frequently used measures is the proportion of days covered (PDC). Research into medication adherence in HF populations requires the collection of this data, which is available in a limited number of studies.

This study aimed to assess adherence to HF medications in patients who had been hospitalised for an acute HF event, and to measure the impact of this adherence on the rate of death and readmissions.

## 2. Materials and Methods

### 2.1. Data Source and Patients

This study was based on data from the FRESH cohort (FREnch Survey on Heart Failure, NCT 01956539). The FRESH cohort enrolled patients aged 18 years and older who were diagnosed with HF during both outpatient consultations and unplanned hospitalisations for acute HF between 18 February 2014 and 31 December 2017. Only patients hospitalised for acute HF were included in this study. Drug prescriptions at discharge were recorded in case report forms of the FRESH study. Drugs which were dispensed from pharmacies were collected in the National Healthcare System Database (NHSD), which contains information on all drugs dispensed in pharmacies, including the date of dispense, strength, and package size. The feasibility and accuracy of linking the FRESH cohort to the national claims database has previously been demonstrated [18].

### 2.2. Drug Adherence

Medication adherence was measured using the proportion of days covered (PDC), which is defined as the number of days on medication divided by the number of days under observation. The PDC was calculated only during the first year after discharge to limit the impact of time and events on adherence. During this period, days in which the patient was in hospital were excluded. A reasonable cut-off point of 80% was used to stratify adherent and non-adherent patients based on predicting subsequent hospitalisation across several highly prevalent chronic diseases [19]. The PDC was calculated for three HF drug classes: beta blockers (BBs), renin-angiotensin system inhibitors (RASis) and mineralocorticoid receptor antagonists (MRAs). Low medication adherence to BBs, RASis and MRAs was defined as PDC <80%. The PDC was capped at 1.0 and multiplied by 100 to obtain a percentage adherence value. Patients were considered to be low adherers when the PDC for at least one drug was below 80%, i.e., if they were taking two drugs but the PDC for one of them was below 80%, they were considered to be non-adherent. An adherence level of 80% or more is thought to be associated with a reduced risk of death in HF, and this is the threshold commonly used in studies of cardiovascular medication adherence [20]. Measurements of medication persistence identified gaps in patients’ timing of prescription refiling that exceeded a predetermined grace period (60 days) after the end of medication supply from the previous refill. Therefore, a patient was classified as non-persistent if he or she did not refill a medication prescription within 60 days. Mean PDC was also measured for six other classes commonly prescribed in HF patients: loop diuretics, calcium channel blockers, statins, anti-coagulants and anti-platelets, proton pump inhibitors, and glucose lowering therapy.

### 2.3. Clinical Events After Discharge

Using the NHSD, we collected data on all-cause mortality, HF, and all-cause hospitalisations. Only first rehospitalisations, involving at least 2 days of hospitalisation, were included. The causes of hospitalisations were classified using the ICD-10 coding system for hospitalisation. The main cause of hospitalisation was HF when the main diagnostic was coded as I50. Patients were followed for at least two years after discharge.

### 2.4. Statistical Analysis

Data are expressed as the mean ± standard deviation, median, and interquartile range or frequency. For analytical purposes, patients were stratified according to PDC using the 80% threshold. Intergroup comparisons were performed using a χ^2^ test for categorical variables, and Student’s *t*-test or the Mann–Whitney test for continuous variables. The Kaplan–Meier actuarial method was used to estimate two-year overall survival based on adherence status. The log-rank test was used to compare survival curves. Univariate and multivariate analyses of all-cause hospitalisation were performed using Cox proportional hazards models. After verifying the proportional hazards assumptions, variables which were significantly associated with hospitalisation in univariate analyses (with a *p*-value threshold of 0.1) were selected for the multivariate model. Age and gender were also included in the adjusted model. A *p*-value ≤ 0.05 was considered statistically significant.

## 3. Results

### 3.1. Population and Baseline Characteristics

**Figure 1:**
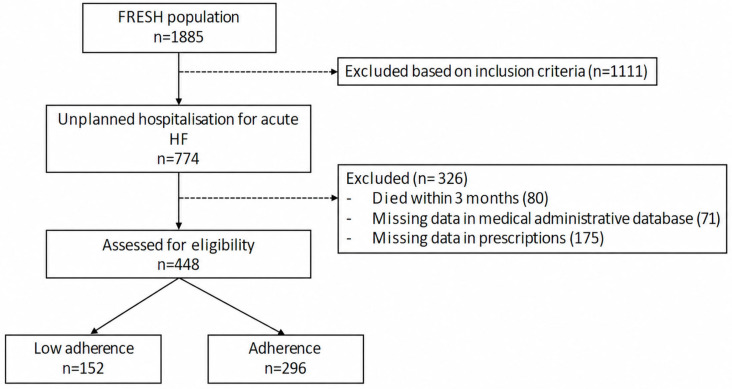
Flowchart of study population selection.

A total of 774 patients from 32 centres were prospectively included (Figure 1). The majority of our cohort population were male (67%), non-smokers (82%), and had a median age of 73 (63–81) years (Table 1). A total of 55% of patients had an ejection fraction ≤40%. Few patients lived alone (17%) and 15% consumed alcohol regularly (>2 drinks per day). Out of 448 patients, 84% received a prescription for loop diuretics, 70% for BBs, 64% for RASis and 30% for MRAs at discharge.

### 3.2. Drug Adherence and Persistence

We divided our population into two groups according to adherence status. A total of 296 patients (66%) were adherent (PDC ≥ 80%), and 152 patients (34%) were low-adherent (PDC < 80%) for at least one of the three HF drugs of interest (BBs, MRAs, RASis). Low-adherent patients had more prescriptions for MRAs (*p* = 0.01) than adherers (Table 1).

**Figure 2:**
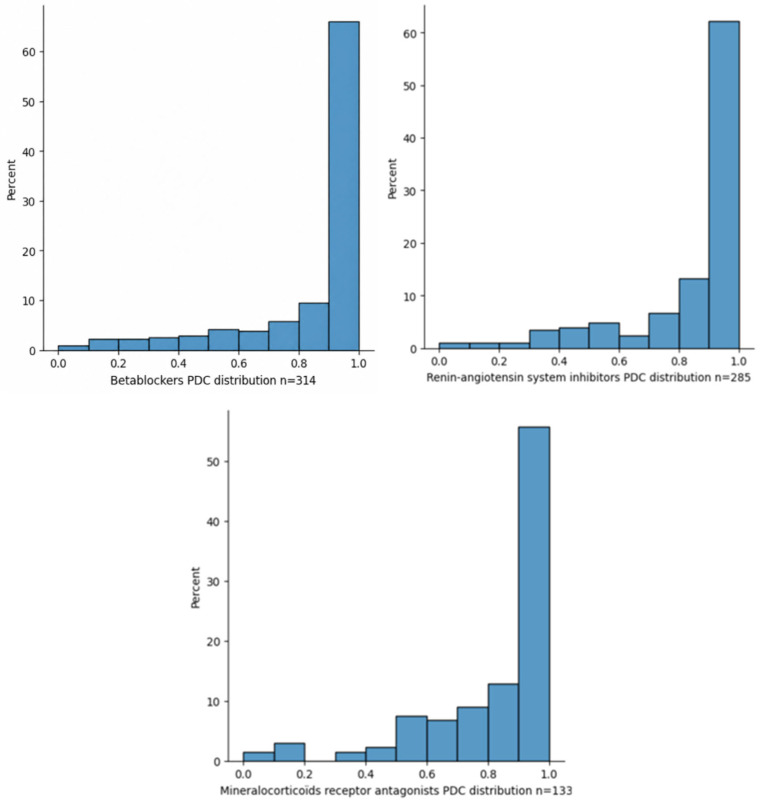
Proportion of days covered (PDC) distribution for beta blockers (BBs), renin-angiotensin system inhibitors (RASis) and mineralocorticoid receptor antagonists (MRAs) one year after discharge.

Figure 2 shows the PDC for each HF drug. Among patients who had been prescribed BB, RASi and MRA prescriptions, 24.5%, 24.6% and 31.5% respectively had a PDC <80% one year after discharge. Adherent patients received about twice the dispensed medication (BBs, RASis, MRAs) per year than low-adherent patients (*p* < 0.001) (Table 2). In terms of target dose, less than a quarter of patients reached 100% of the target dose regardless of group (adherent or not), but the majority of patients received at least ≥50% of the target dose. Regarding the prescription of HF drugs at discharge, 50% of patients had monotherapy, 36.6% had dual therapy, and 13.4% had triple therapy (Supplementary Data—Table S1). During follow-up, there was a gradual decrease in persistence (Supplementary Figure S1), and one year after discharge, the proportion of patients who were no longer taking the medication was between 20.3% for beta blockers and 36.3% for dual HF therapy (Supplementary Table S1).

Regarding other drugs we analysed in the health database, the rates of low adherence were particularly high, from 34% for statins up to 49% and 52% for antithrombotic drugs and proton pump inhibitors, respectively (Supplementary Table S2).

### 3.3. Association Between Medication Adherence and Outcomes After Discharge

**Figure 3:**
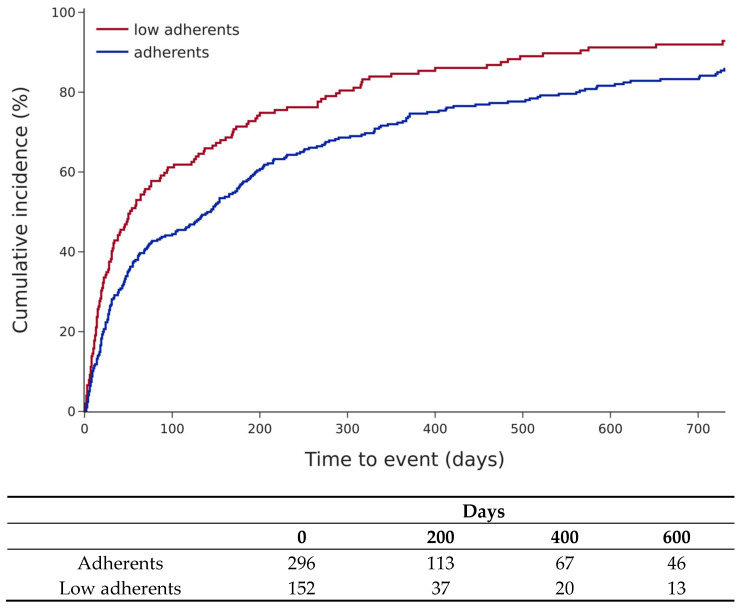
Cumulative incidence of all-cause hospitalisations according to adherent status (log-rank test, *p* = 0.0004).

A total of 381 (85%) patients were hospitalised, 218 (48.7% for HF) for any cause. Patients with low adherence had significantly more hospitalisations for any cause (hazard ratio (HR) 1.45 (95% confidence interval: 1.18–1.8, *p* < 0.01), Figure 3). In Cox multivariate models, only adherence status and diabetes were significantly associated with all-cause hospitalisations (Table 3).

**Figure 4:**
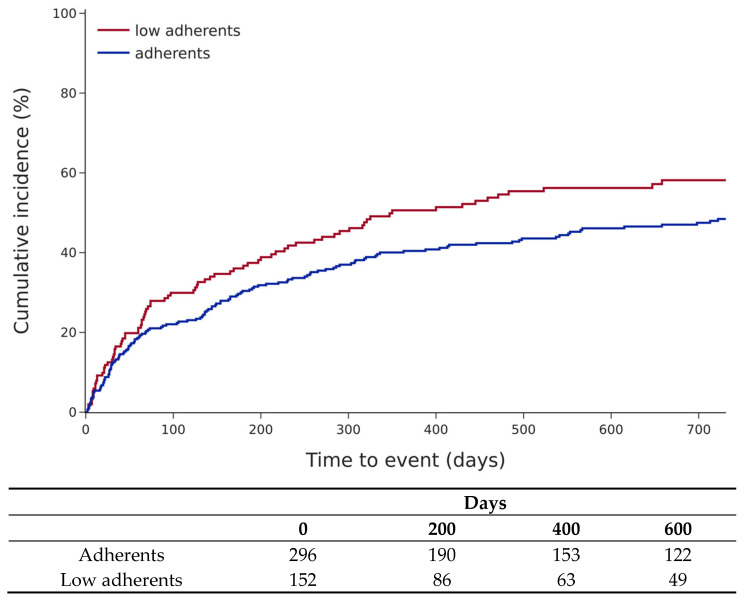
Cumulative incidence of HF rehospitalisations according to adherent status (log-rank test, *p* = 0.04).

Patients with low adherence also had significantly more HF hospitalisations (HR 1.33 (95% confidence interval: 1.01–1.74, *p* = 0.04), Figure 4), but low-adherent status was not significantly associated with HF hospitalisations in the Cox multivariate model (Table 4).

**Figure 5:**
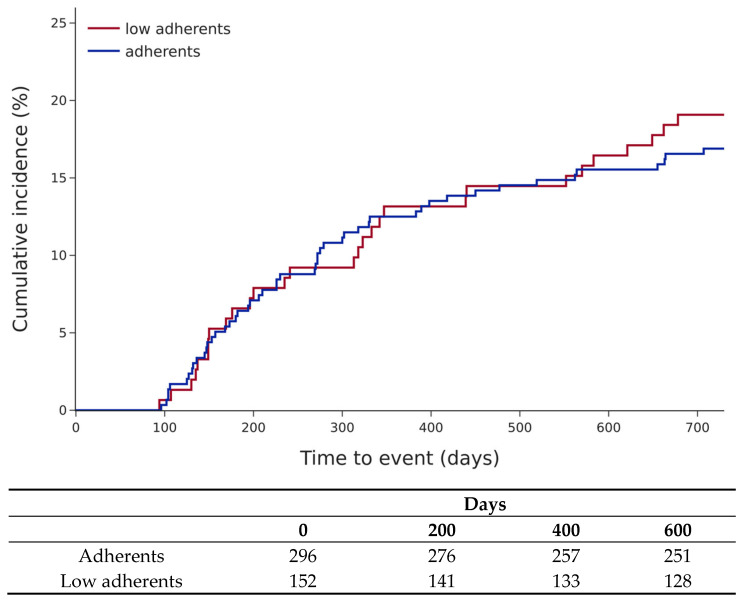
Cumulative incidence of all-cause death according to adherent status (log-rank test, *p* = 0.6).

A total of 79 patients (18%) died; there was no significant difference in mortality between the two groups (19.1% versus 16.9%) (Figure 5).

## 4. Discussion

This study describes medication adherence and non-persistence profiles among patients discharged after hospitalisation for HF. Our results highlight the high rate of low adherence—around 34% for HF drugs—during follow-up, regardless of the drug. Importantly, low adherence is associated with poor outcome. Indeed, we found that the rates of all-cause and HF-related rehospitalisation rates were significantly higher in the low-adherent group. Multivariate analysis identified low adherence as an independent risk factor for rehospitalisation. Moreover, almost 30% of patients were not persistent in taking HF drugs a year after discharge.

There are several methods for measuring adherence [17]. The PDC method is the most frequently used, with a cut-off point of 80% used to define patients as having low adherence. Qin et al. examined 4234 HF patients and showed that PDC provided a more conservative estimate of adherence compared to two traditional methods—medication possession ratio and modified medication possession ratio [21]. Considering our cohort of 448 patients, we measured PDC for HF therapies without dividing them into subgroups (mono, dual and triple therapy) and obtained a global PDC. Therefore, comparison with other studies may not be appropriate, but the adherence profiles tend to be similar. Odegaard at al., in a cohort of 54,899 Norwegian HF patients, calculated a PDC for each HF drug class over a year. They found that over 80% of patients were adherent for BBs and RASis, and 61% for MRAs. For dual and triple therapy, the PDC decreased to 42% and 5%, respectively [22]. Scalvini et al., in large population of 100,422 Italian HF patients, showed that adherence to medication was particularly low with triple HF therapy: PDC was 42 ± 28% [23]. Our study was conducted between 2014 and 2017 at a time when triple therapy was recommended for patients with reduced EF. Since then, sodium-glucose cotransporter 2 inhibitors (SGLT2is) have emerged and were commercialised in France from 2020, leading to a quadruple therapy, which could increase low adherence. In fact, polypharmacy could be an additional factor linked to poor adherence. In our study, patients in the low-adherent group had significantly more polymedication (≥5 drugs), which highlights the detrimental role of polymedication on adherence. Pasina et al. also showed that an increasing number of drugs prescribed at hospital discharge was correlated with non-adherence, and that a high percentage of patients did not understand the purpose of their medications [24]. Less expectedly, MRA prescription was also associated with low adherence, but the reasons behind this result are much less clear than those behind the association with polymedication. Low medication adherence is a real, complex problem influenced by several patient-related factors ranging from patient education to socio-economic factors and the healthcare system. In France, HF patients have full medication coverage, which excludes issues related to drug costs not related to their condition. Adherence to medication is a multifaceted issue that requires a multidimensional approach to improve patient outcomes. A recent study has shown that a low health-related quality of life (as evaluated by EQ-5D) and depression were associated with the non-persistence of medication [25]. A recent systematic review of 516,244 HF patients has shown a statistical relationship between depression and medication adherence, which was partially explained by a lack of motivation, fatigue and mental illness [26].

A high percentage of patients do not have a full understanding of their condition, which could contribute to low adherence [24]. It is therefore crucial to support these patients by improving therapeutic education within multidisciplinary teams, including different specialists (cardiologists, nurses, pharmacists and general practitioners) to reinforce messages. Several studies have been carried out to highlight the role of multidisciplinary teams in supporting patients and improving medication adherence [27,28,29,30,31]. Several randomised controlled clinical trials have shown improvements in medication adherence and quality of life in HF patients [32,33,34]. A medication review conducted by pharmacists provides recommendations to improve treatment safety and effectiveness. The pharmaceutical consultation improved HF medication adherence through patient education on proper use and interactions. Technological tools can further support adherence, especially at hospital discharge [35,36].

This low level of adherence, which is associated with misunderstandings about the impact of the disease, increases rehospitalisation rates and mortality. Several studies have found an association between non-adherence and non-persistence, which is linked to a higher risk of rehospitalisation and mortality [14,20,23,37]. Our study showed that the rates of all-cause and HF rehospitalisation at two years were lower in adherent patients than in patients with low adherence. The absence of impact on mortality in our study could be due to the exclusion of patients who were the most severely ill and died within three months post-discharge, as well as our study having a shorter follow-up than the study of Gislason et al. [14]. On the other hand, a meta-analysis showed that incremental adherence thanks to interventions such as medication and disease education, self-monitoring and self-management have reduced readmission rates and HF mortality [37], reinforcing the crucial role of HF patient education in improving prognosis [14].

Adherence rates vary widely depending on the disease. For example, in a study of atrial fibrillation, 25% of the 33,311 patients treated with a direct anti-coagulant were non-adherent [38]. A meta-analysis that included more than 30 observational studies involving over 500,000 patients found that, after one year, up to 30% of patients were non-adherent to their treatment, with mean adherence scores of 77% and 74% at six and 12 months, respectively. This confirms limited persistence, even in a controlled clinical trial setting [39].

### Limitations

Our study was conducted prior to the widespread use of SGLT2 inhibitors in HF, but it is unlikely that the addition of this new therapeutic class will positively impact compliance, as observed in our study. The method we used to assess medication adherence, which was based on pharmacy dispensing records, does not guarantee that patients actually took their medications as prescribed. Excluding patients who died within three months of discharge may have had an adverse effect on the analysis of the association between adherence and mortality. Furthermore, estimating adherence and persistence from prescription refill data does not consider the underlying reasons why some prescriptions were not filled.

## 5. Conclusions

This study has shown the importance of the problem of low adherence and non-persistence to medication by HF patients and its impact on outcome. This issue deserves to be given greater attention, particularly after hospitalisation. The evaluation of therapeutic strategies including HF team, telemonitoring, pharmacists, or multidisciplinary networks must include their impact on treatment compliance.

## Figures and Tables

**Table 1:** Baseline characteristics according to adherent status.

	Adherent *n* = 296	Low-Adherent *n* = 152	Total *n* = 448	Missing Data (%)	*p*
Age (median IQR)	73 (63–81)	75 (64–81)	73 (63–81)	0	0.49
Men (*n*, %)	193 (65)	107 (70)	300 (67)	0	0.32
BMI kg/m^2^ (mean ± SD)	28 ± 6	27 ± 6	27 ± 6	7	0.05
GFR mL/min (median, IQR)	55 (40–71)	52 (41–73)	53 (40–71)	0.4	0.43
Graduate (*n*, %)	40 (26.1)	19 (23.8)	59 (25)	48	0.81
Smoking status (*n*, %)	43 (18)	22 (18)	65 (18)	19	1
Smoking cessation (*n*, %)	109 (45)	55 (45)	164 (45)	19	1
Living alone (*n*, %)	45 (17)	22 (17)	67 (17)	14	1
Alcohol >2 drinks a day (*n*, %)	27 (15)	15 (15)	42 (15)	36	1
Comorbidities (*n*, %)					
Atrial fibrillation	132 (49)	81 (59)	213 (53)	9	0.07
Diabetes	105 (36)	44 (29)	149 (33)	0	0.2
Hypertension	199 (69)	97 (66)	296 (67)	2	0.59
Dyslipidaemia	147 (51)	65 (45)	212 (49)	3	0.25
Cerebrovascular event	40 (14)	27 (18)	67 (15)	3	0.34
Depression	23 (8)	14 (10)	37 (9)	5	0.73
Heart failure causes (*n*, %)					0.22
Ischemic	98 (33)	51 (34)	149 (33)	0	
Valvular	21 (7)	18 (12)	39 (9)	0	
Other	177 (60)	83 (55)	260 (58)	0	
De novo heart failure (*n*, %)	126 (43)	62 (41)	188 (42)	1	0.79
HF hospitalisation within 2 months	25 (8)	12 (8)	37 (8)		0.98
Ejection fraction (*n*, %)					
≤40% (%)	160 (54)	85 (56)	245 (55)	2	0.84
41–49% (%)	30 (10)	19 (13)	49 (11)	2	0.57
≥50% (%)	104 (35)	48 (32)	152 (34)	2	0.49
Drugs at discharge (*n*, %)					
Loop diuretics	249 (84)	119 (78)	368 (82)	0	0.16
RASis	182 (62)	103 (68)	285 (64)	0	0.23
Beta blockers	204 (69)	110 (72)	314 (70)	0	0.52
MRAs	76 (26)	57 (38)	133 (30)	0	0.01
Calcium channel blockers	38 (13)	21 (14)	59 (13)	0	0.89
Polymedication ≥ 5 (FDAD)	241 (81)	109 (72)	350 (78)	0	0.03
Polymedication ≥ 10 (FDAD)	87 (29)	34 (22)	121 (27)	0	0.14

IQR: Interquartile range; SD: standard derivation; BMI: body mass index; GFR: glomerular filtration rate; RASis: renin-angiotensin system inhibitors; MRAs: mineralocorticoid receptor antagonists; FDAD: first dispensation after discharge.

**Table 2:** Dispensed medication and target dose among adherent status within one year after discharge.

	Adherent	Low-Adherent	Total	*p*
Dispensed number (mean ± SD) *				
BBs (*n* = 281)	12 ± 3	6.8 ± 3.9	10.2 ± 4.2	<0.001
RASis (*n* = 250)	11± 3.2	6.5 ± 3.6	9.4 ± 4	<0.001
MRAs (*n* = 111)	11 ± 2.6	6.3 ± 3.2	9.1 ± 3.4	<0.001
Proportion ≥ 50% target dose (*n*, %) *				
BBs (*n* = 281)	95 (51.4)	49 (51)	144 (51,2)	0.96
RASis (*n* = 250)	86 (54)	44 (48.4)	130 (52)	0.3
MRAs (*n* = 111)	66 (100)	45 (100)	111 (100)	1.0
Proportion 100% target dose (*n*, %) *				
BBs (*n* = 281)	38 (20.5)	11 (11.5)	49 (17.4)	0.07
RASis (*n* = 250)	35 (22)	20 (22)	55 (22)	1
MRAs (*n* = 111)	17 (25.8)	12 (26.7)	29 (26.1)	1

* *n* = 391 patients were alive after 1 year of follow-up.

**Table 3:** Univariate and multivariate Cox regression analyses of risk factors for all-cause hospitalisations.

	Univariate Analysis		Multivariate Analysis	
	HR [95% CI]	*p*	HR [95% CI]	*p*
Gender	0.96 [0.78–1.19]	0.72		
Age	1.01 [1.00–1.01]	0.17		
BMI	1.01 [0.99–1.03]	0.39		
Living alone	0.88 [0.66–1.17]	0.37		
Active smoker	0.75 [0.55–1.01]	0.06	0.83 [0.56–1.25]	0.38
Ejection fraction < 40%	1.04 [0.85–1.27]	0.71		
Ejection fraction 41–49%	0.91 [0.66–1.25]	0.55		
Ejection fraction ≥ 50%	1.01 [0.81–1.24]	0.98		
Alcohol	0.65 [0.45–0.94]	0.02	0.70 [0.44–1.11]	0.13
Diabetes	1.46 [1.18–1.80]	<0.001	1.42 [1.04–1.95]	0.03
Hypertension	0.95 [0.77–1.19]	0.67		
Atrial fibrillation	1.22 [0.99–1.51]	0.06	1.10 [0.82–1.48]	0.50
Stroke	1.25 [0.95–1.64]	0.11		
LVEF	0.99 [0.98–1.00]	0.27		
Depression	1.04 [0.72–1.49]	0.84		
eGFR < 60 mL/min/1.73 m^2^	1.25 [1.01–1.54]	0.04	1.04 [0.77–1.39]	0.81
Adherence status	0.68 [0.56–0.85]	<0.001	0.54 [0.4–0.73]	<0.001

HR: hazard ratio; BMI: body mass index; CI: confidence interval; LVEF: left ventricular ejection fraction; eGFR: estimated glomerular filtration rate.

**Table 4:** Univariate and multivariate Cox regression analyses of risk factors for HF hospitalisation.

	Univariate Analysis		Multivariate Model	
	HR [95% CI]	*p*	HR [95% CI]	*p*
Sex	0.92 [0.69–1.22]	0.56		
Age	1.01 [0.99–1.02]	0.56		
BMI	1.01 [0.98–1.03]	0.62		
Living alone	0.76 [0.50–1.14]	0.18		
Active smoker	0.4 [0.24–0.65]	<0.001	0.57 [0.33–0.99]	0.04
Ejection fraction < 40%	1.12 [0.85–1.46]	0.43		
Ejection fraction 41–49%	1.08 [0.72–1.63]	0.70		
Ejection fraction ≥ 50%	0.85 [0.64–1.14]	0.28		
Alcohol	0.55 [0.32–0.94]	0.03	0.55 [0.28–1.07]	0.08
Diabetes	1.24 [0.94–1.63]	0.13		
Hypertension	0.92 [0.69–1.22]	0.56		
Atrial fibrillation	1.19 [0.89–1.57]	0.24		
Stroke	1.78 [1.28–2.47]	<0.001	1.96 [1.29–2.98]	0.001
LVEF	0.99 [0.98–1]	0.09	0.99 [0.98–1]	0.03
Depression	0.92 [0.57–1.53]	0.78		
eGFR < 60 mL/min	1.98 [1.47–2.66]	<0.001	1.79 [1.22–2.62]	0.003
Adherence status	0.75 [0.57–0.99]	0.04	0.74 [0.52–1.06]	0.1

HR: hazard ratio; CI: confidence interval; BMI: body mass index; LVEF: left ventricular ejection fraction; eGFR: estimated glomerular filtration rate.

## Data Availability

The data underlying this article will be shared upon reasonable request to the corresponding author.

## Supplementary Materials

The following supporting information can be downloaded at: https://www.mdpi.com/article/10.3390/jcm15093483/s1, Table S1: Lack of persistence during the first years after discharge. Table S2: Proportion of low adherent patients (PDC<80%) for commonly used drug classes. Figure S1: Lack of persistence for HF drugs during the first year after discharge.

## Abbreviations

The following abbreviations are used in this manuscript.

HF	Heart failure
PDC	Proportion of days covered
BBs	Beta blockers
RASis	Renin-angiotensin system inhibitors
MRAs	Mineralocorticoid receptor antagonists
HR	Hazard ratio
LVEF	Left ventricular ejection fraction
eGFR	Estimated glomerular filtration rate

## References

[1] Bragazzi N.L., Zhong W., Shu J., Abu Much A., Lotan D., Grupper A., Younis A., Dai H. (2021). Burden of Heart Failure and Underlying Causes in 195 Countries and Territories from 1990 to 2017. Eur. J. Prev. Cardiol..

[2] Savarese G., Becher P.M., Lund L.H., Seferovic P., Rosano G.M.C., Coats A.J.S. (2022). Global Burden of Heart Failure: A Comprehensive and Updated Review of Epidemiology. Cardiovasc. Res..

[3] McDonagh T.A., Metra M., Adamo M., Gardner R.S., Baumbach A., Böhm M., Burri H., Butler J., Čelutkienė J., Chioncel O. (2023). 2023 Focused Update of the 2021 ESC Guidelines for the Diagnosis and Treatment of Acute and Chronic Heart Failure. Eur. Heart J..

[4] Vaduganathan M., Claggett B.L., Jhund P.S., Cunningham J.W., Pedro Ferreira J., Zannad F., Packer M., Fonarow G.C., McMurray J.J.V., Solomon S.D. (2020). Estimating Lifetime Benefits of Comprehensive Disease-Modifying Pharmacological Therapies in Patients with Heart Failure with Reduced Ejection Fraction: A Comparative Analysis of Three Randomised Controlled Trials. Lancet.

[5] Zannad F., McMurray J.J.V., Krum H., van Veldhuisen D.J., Swedberg K., Shi H., Vincent J., Pocock S.J., Pitt B. (2011). EMPHASIS-HF Study Group. Eplerenone in Patients with Systolic Heart Failure and Mild Symptoms. N. Engl. J. Med..

[6] Myhre P.L., Vaduganathan M., Claggett B., Packer M., Desai A.S., Rouleau J.L., Zile M.R., Swedberg K., Lefkowitz M., Shi V. (2019). B-Type Natriuretic Peptide During Treatment with Sacubitril/Valsartan: The PARADIGM-HF Trial. J. Am. Coll. Cardiol..

[7] McMurray J.J.V., Solomon S.D., Inzucchi S.E., Køber L., Kosiborod M.N., Martinez F.A., Ponikowski P., Sabatine M.S., Anand I.S., Bělohlávek J. (2019). Dapagliflozin in Patients with Heart Failure and Reduced Ejection Fraction. N. Engl. J. Med..

[8] Packer M., Butler J., Zannad F., Filippatos G., Ferreira J.P., Pocock S.J., Carson P., Anand I., Doehner W., Haass M. (2021). Effect of Empagliflozin on Worsening Heart Failure Events in Patients with Heart Failure and Preserved Ejection Fraction: EMPEROR-Preserved Trial. Circulation.

[9] Solomon S.D., McMurray J.J.V., Claggett B., de Boer R.A., DeMets D., Hernandez A.F., Inzucchi S.E., Kosiborod M.N., Lam C.S.P., Martinez F. (2022). Dapagliflozin in Heart Failure with Mildly Reduced or Preserved Ejection Fraction. N. Engl. J. Med..

[10] Anker S.D., Butler J., Filippatos G., Ferreira J.P., Bocchi E., Böhm M., Brunner-La Rocca H.-P., Choi D.-J., Chopra V., Chuquiure-Valenzuela E. (2021). Empagliflozin in Heart Failure with a Preserved Ejection Fraction. N. Engl. J. Med..

[11] Wirtz H.S., Sheer R., Honarpour N., Casebeer A.W., Simmons J.D., Kurtz C.E., Pasquale M.K., Globe G. (2020). Real-World Analysis of Guideline-Based Therapy After Hospitalization for Heart Failure. J. Am. Heart Assoc..

[12] Tsigkas G., Apostolos A., Aznaouridis K., Despotopoulos S., Chrysohoou C., Naka K.K., Davlouros P. (2022). Real-World Implementation of Guidelines for Heart Failure Management: A Systematic Review and Meta-Analysis. Hell. J. Cardiol..

[13] Chang H.-Y., Wang C.-C., Wei J., Chang C.-Y., Chuang Y.-C., Huang C.-L., Chong E., Lin J.-L., Mar G.-Y., Chan K.-C. (2017). Gap between Guidelines and Clinical Practice in Heart Failure with Reduced Ejection Fraction: Results from TSOC-HFrEF Registry. J. China Med. Assoc..

[14] Gislason G.H., Rasmussen J.N., Abildstrom S.Z., Schramm T.K., Hansen M.L., Buch P., Sørensen R., Folke F., Gadsbøll N., Rasmussen S. (2007). Persistent Use of Evidence-Based Pharmacotherapy in Heart Failure Is Associated with Improved Outcomes. Circulation.

[15] Carnicelli A.P., Li Z., Greiner M.A., Lippmann S.J., Greene S.J., Mentz R.J., Hardy N.C., Blumer V., Shen X., Yancy C.W. (2021). Sacubitril/Valsartan Adherence and Postdischarge Outcomes Among Patients Hospitalized for Heart Failure with Reduced Ejection Fraction. JACC Heart Fail..

[16] Vrijens B., De Geest S., Hughes D.A., Przemyslaw K., Demonceau J., Ruppar T., Dobbels F., Fargher E., Morrison V., Lewek P. (2012). A New Taxonomy for Describing and Defining Adherence to Medications. Br. J. Clin. Pharmacol..

[17] Hess L.M., Raebel M.A., Conner D.A., Malone D.C. (2006). Measurement of Adherence in Pharmacy Administrative Databases: A Proposal for Standard Definitions and Preferred Measures. Ann. Pharmacother..

[18] Logeart D., Damy T., Doublet M., Salvat M., Tribouilloy C., Bauer F., Eicher J.-C., Picard F., Roul G., Trochu J.-N. (2023). Feasibility and Accuracy of Linking a Heart Failure Registry to the National Claims Database Using Indirect Identifiers. Arch. Cardiovasc. Dis..

[19] Karve S., Cleves M.A., Helm M., Hudson T.J., West D.S., Martin B.C. (2009). Good and Poor Adherence: Optimal Cut-Point for Adherence Measures Using Administrative Claims Data. Curr. Med. Res. Opin..

[20] Fitzgerald A.A., Powers J.D., Ho P.M., Maddox T.M., Peterson P.N., Allen L.A., Masoudi F.A., Magid D.J., Havranek E.P. (2011). Impact of Medication Nonadherence on Hospitalizations and Mortality in Heart Failure. J. Card. Fail.

[21] Qin X., Hung J., Knuiman M.W., Briffa T.G., Teng T.-H.K., Sanfilippo F.M. (2020). Comparison of Medication Adherence Measures Derived from Linked Administrative Data and Associations with Mortality Using Restricted Cubic Splines in Heart Failure Patients. Pharmacoepidemiol. Drug Saf..

[22] Ødegaard K.M., Lirhus S.S., Melberg H.O., Hallén J., Halvorsen S. (2023). Adherence and Persistence to Pharmacotherapy in Patients with Heart Failure: A Nationwide Cohort Study, 2014–2020. ESC. Heart Fail..

[23] Scalvini S., Bernocchi P., Villa S., Paganoni A.M., La Rovere M.T., Frigerio M. (2021). Treatment Prescription, Adherence, and Persistence after the First Hospitalization for Heart Failure: A Population-Based Retrospective Study on 100,785 Patients. Int. J. Cardiol..

[24] Pasina L., Brucato A.L., Falcone C., Cucchi E., Bresciani A., Sottocorno M., Taddei G.C., Casati M., Franchi C., Djade C.D. (2014). Medication Non-Adherence among Elderly Patients Newly Discharged and Receiving Polypharmacy. Drugs Aging.

[25] Rasmussen A.A., Wiggers H., Jensen M., Berg S.K., Rasmussen T.B., Borregaard B., Thrysoee L., Thorup C.B., Mols R.E., Larsen S.H. (2021). Patient-Reported Outcomes and Medication Adherence in Patients with Heart Failure. Eur. Heart J. Cardiovasc. Pharmacother..

[26] Poletti V., Pagnini F., Banfi P., Volpato E. (2022). The Role of Depression on Treatment Adherence in Patients with Heart Failure-a Systematic Review of the Literature. Curr. Cardiol. Rep..

[27] Zheng J., Mednick T., Heidenreich P.A., Sandhu A.T. (2023). Pharmacist- and Nurse-Led Medical Optimization in Heart Failure: A Systematic Review and Meta-Analysis. J. Card. Fail.

[28] Jasińska-Stroschein M., Waszyk-Nowaczyk M. (2023). Multidimensional Interventions on Supporting Disease Management for Hospitalized Patients with Heart Failure: The Role of Clinical and Community Pharmacists. J. Clin. Med..

[29] Jarab A.S., Al-Qerem W.A., Hamam H.W., Alzoubi K.H., Abu Heshmeh S.R., Mukattash T.L., Alefishat E. (2023). Medication Adherence and Its Associated Factors Among Outpatients with Heart Failure. Patient Prefer. Adherence.

[30] Granger B.B., Ekman I., Hernandez A.F., Sawyer T., Bowers M.T., DeWald T.A., Zhao Y., Levy J., Bosworth H.B. (2015). Results of the Chronic Heart Failure Intervention to Improve MEdication Adherence Study: A Randomized Intervention in High-Risk Patients. Am. Heart J..

[31] Berardinelli D., Conti A., Hasnaoui A., Casabona E., Martin B., Campagna S., Dimonte V. (2024). Nurse-Led Interventions for Improving Medication Adherence in Chronic Diseases: A Systematic Review. Healthcare.

[32] Wang L., Zhao Y., Han L., Zhang H., Chen H., Liu A., Yu J., Fu R., Duan L., An F. (2024). Pharmacist-Led Management Model and Medication Adherence Among Patients with Chronic Heart Failure: A Randomized Clinical Trial. JAMA Netw. Open.

[33] Schulz M., Griese-Mammen N., Anker S.D., Koehler F., Ihle P., Ruckes C., Schumacher P.M., Trenk D., Böhm M., Laufs U. (2019). Pharmacy-Based Interdisciplinary Intervention for Patients with Chronic Heart Failure: Results of the PHARM-CHF Randomized Controlled Trial. Eur. J. Heart Fail.

[34] Schumacher P.M., Becker N., Tsuyuki R.T., Griese-Mammen N., Koshman S.L., McDonald M.A., Bouvy M., Rutten F.H., Laufs U., Böhm M. (2021). The Evidence for Pharmacist Care in Outpatients with Heart Failure: A Systematic Review and Meta-Analysis. ESC. Heart Fail.

[35] Cheng C., Donovan G., Al-Jawad N., Jalal Z. (2023). The Use of Technology to Improve Medication Adherence in Heart Failure Patients: A Systematic Review of Randomised Controlled Trials. J. Pharm. Policy Pract..

[36] FarzanehRad A., Allahbakhshian A., Gholizadeh L., Khalili A.F., Hasankhani H. (2024). Randomized Comparison of the Effects of Tailored Text Messaging versus Pillbox Organizers on Medication Adherence of Heart Failure Patients. BMC Cardiovasc. Disord..

[37] Ruppar T.M., Cooper P.S., Mehr D.R., Delgado J.M., Dunbar-Jacob J.M. (2016). Medication Adherence Interventions Improve Heart Failure Mortality and Readmission Rates: Systematic Review and Meta-Analysis of Controlled Trials. J. Am. Heart Assoc..

[38] Perreault S., de Denus S., White-Guay B., Côté R., Schnitzer M.E., Dubé M.-P., Dorais M., Tardif J.-C. (2020). Oral Anticoagulant Prescription Trends, Profile Use, and Determinants of Adherence in Patients with Atrial Fibrillation. Pharmacotherapy.

[39] Salmasi S., Loewen P.S., Tandun R., Andrade J.G., De Vera M.A. (2020). Adherence to Oral Anticoagulants among Patients with Atrial Fibrillation: A Systematic Review and Meta-Analysis of Observational Studies. BMJ Open.

