# Supplementary material for: Drug Adherence After Hospitalisation for Heart Failure: What Have We Learned from a French Survey?
Source: J Clin Med. 2026 May 2;15(9):3483. doi: 10.3390/jcm15093483 (PMC13163470; doi:10.3390/jcm15093483)
Supplement: Supplementary file 1 [file jcm-15-03483-s001.zip › jcm-4225270-supplementary.pdf]

Supplementary Data

Supplementary Table S1: Lack of persistence during the first years after discharge.

HF: Heart Failure, BB: beta-blockers, RASi: renin-angiotensin system inhibitors, MRA: mineralocorticoid receptor antagonists

HF Medication	One year (%)
Monotherapy (n=333)<br>- BB (n=109)<br>- RASi (n=79)<br>- MRA (n=36)	 20.3<br>33.4<br>26.9
Dual therapy (n=164)	36.3
Triple therapy (n=60)	30.6

Supplementary Table S2: Proportion of low adherent patients (PDC<80%) for commonly used drug classes.

PDC: proportion of days covered; HF: Heart Failure; BB: beta blocker; RASi: renin-angiotensin system inhibitors, MRA: mineralocorticoid receptor antagonists, PPI: proton pump inhibitors

**Drugs**	**PDC (mean ± SD)**	**PDC < 80% (%)**
PPI (n=317)	0.6 ± 0.4	52.0
Anti-coagulant/anti-platelets (n=402)	0.7 ± 0.3	49.0
Triple HF therapy (n=60)	0.8 ± 0.3	43.3
Glucose Lowering therapy (n=114)	0.7 ± 0.3	42.0
Calcium channel blocker (n=119)	0.8 ± 0.5	41.0
Dual HF therapy (n=164)	0.8 ± 0.3	40.2
Statin (n=259)	0.8 ± 0.3	34.0
MRA (n=133)	0.8± 0.2	31.5
Loop diuretic (n=427)	0.8 ± 0.3	26.0
RASi (n=285)	0.8 ± 0.2	24.6
BB (n=314)	0.8 ± 0.2	24.5

Supplementary Figure S1: Heart failure treatment no persistence during the first year after discharge

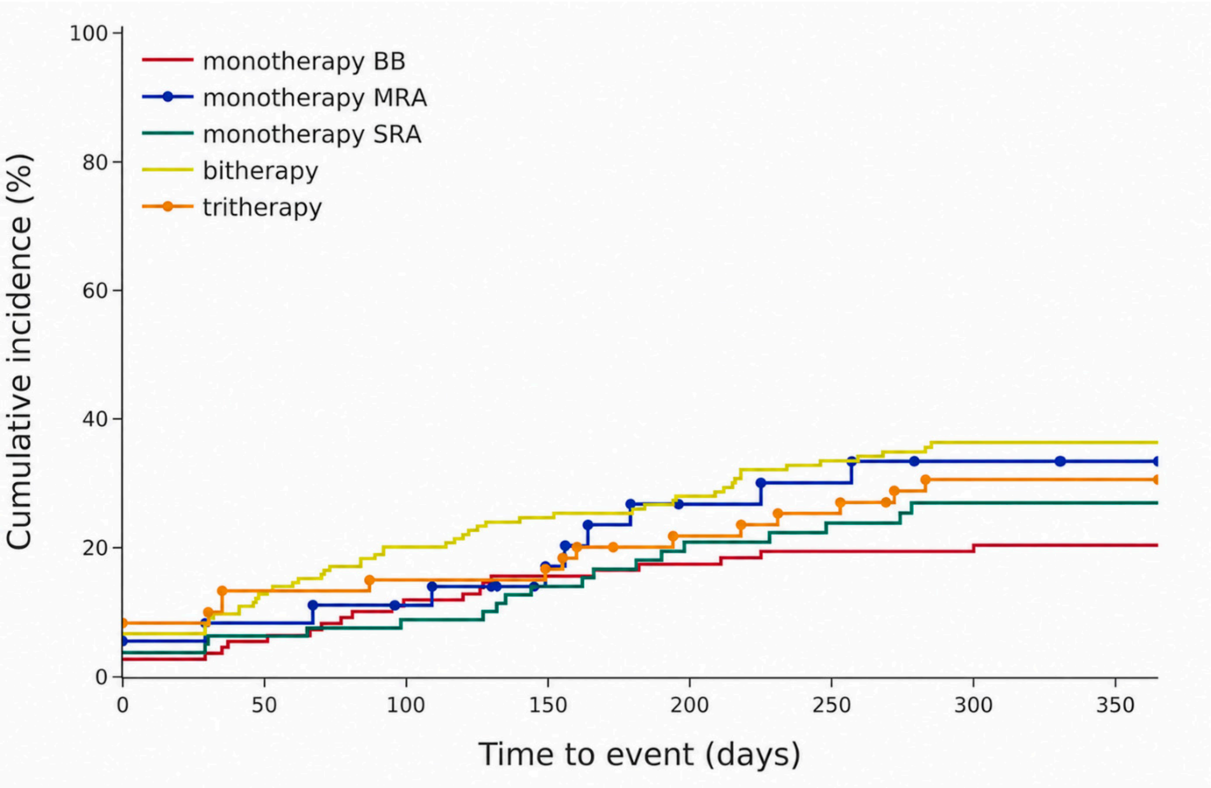

BB: beta blocker; RASi: renin-angiotensin system inhibitors, MRA: mineralocorticoid receptor antagonists
